# Supplementary material for: Downregulation of miR-122-5p Activates Glycolysis via PKM2 in Kupffer Cells of Rat and Mouse Models of Non-Alcoholic Steatohepatitis
Source: Int J Mol Sci. 2022 May 7;23(9):5230. doi: 10.3390/ijms23095230 (PMC9101520; doi:10.3390/ijms23095230)
Supplement: Supplementary file 1 [file ijms-23-05230-s001.zip › Supplementary_Figure.pdf]

International Journal of *Molecular Sciences*

MDPI

Article

Downregulation of miR-122-5p Activates Glycolysis via PKM2 in Kupffer Cells of Rat and Mouse Models of Non-Alcoholic Steatohepatitis

Supplementary Figures

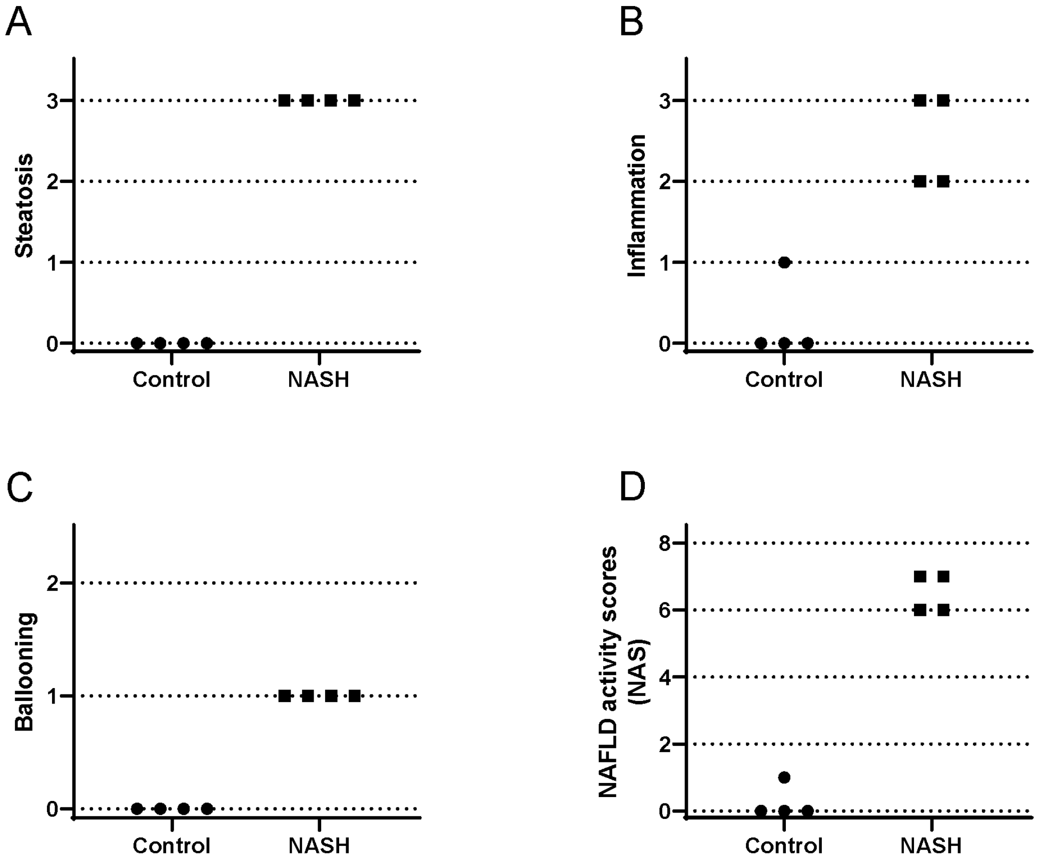

Figure S1. Progress evaluation of steatohepatitis between SHRSP5/Dmcr rats fed with the standard diet (SD) or high-fat and high-cholesterol (HFC) diet for 8 weeks. (A) Steatosis, (B) inflammation, (C) ballooning, and (D) NAFLD activity scores (NAS) were measured.

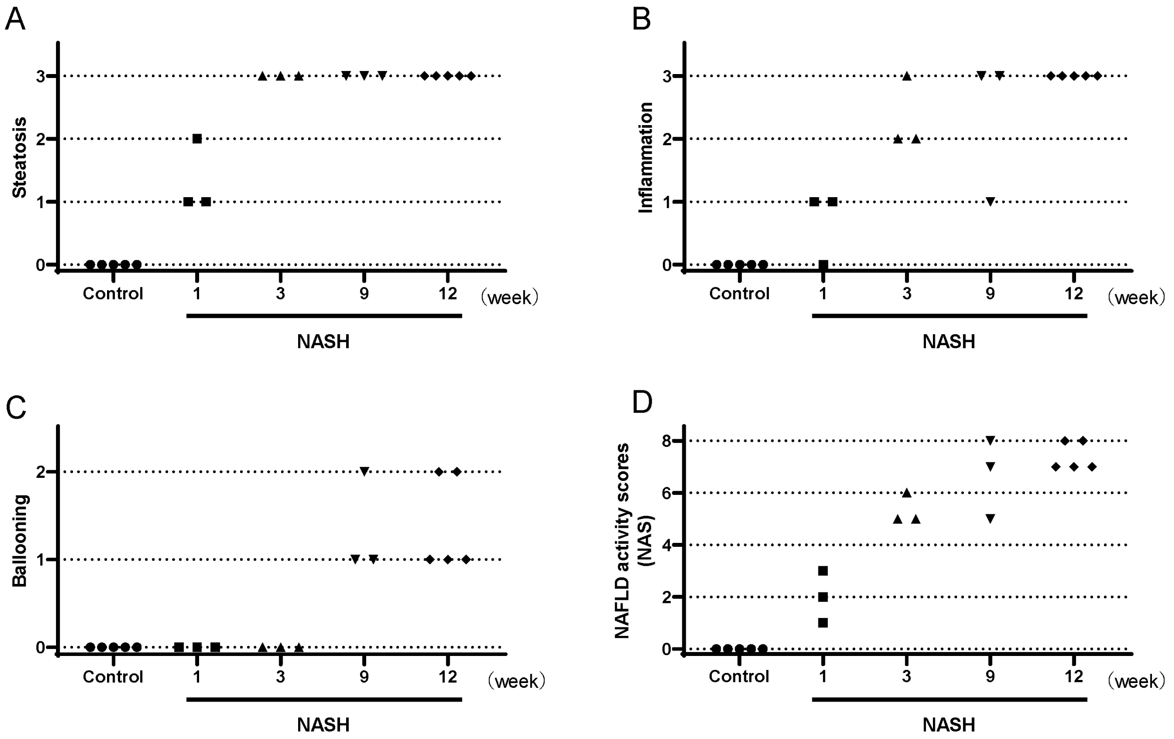

Figure S2. Progress evaluation of steatohepatitis between C57BL/6J mice fed with the standard diet (SD) for 12 weeks or choline-deficient, L-amino-acid-defined, high-fat diet (CDAHFD) for 1, 3, 9, or 12 weeks. (A) Steatosis, (B) inflammation, (C) ballooning, and (D) NAFLD activity scores (NAS) were measured.
